# Genetic Diversity and Population Structure of Rice Varieties Cultivated in Temperate Regions

**DOI:** 10.1186/s12284-016-0130-5

**Published:** 2016-10-20

**Authors:** Juan L. Reig-Valiente, Juan Viruel, Ester Sales, Luis Marqués, Javier Terol, Marta Gut, Sophia Derdak, Manuel Talón, Concha Domingo

**Affiliations:** 1 Centro de Genómica, Instituto Valenciano de Investigaciones Agrarias, Carretera CV 315 Km 10,7 (Carretera Moncada – Náquera Km 4.5), 46113 Moncada, Spain; 2 Dpto. Biología Vegetal y Ecología, SGI Herbario – Universidad de Sevilla, Edif. Celestino Mutis, Av. Reina Mercedes s/n, 41012 Sevilla, Spain; 3 Institut Méditerranéen de Biodiversité et d’Ecologie Marine et Continentale (IMBE), Aix Marseille Université, Chemin de la Batterie des Lions, 13007 Marseille, France; 4 Dpto. Ciencias Agrarias y del Medio Natural, Escuela Politécnica Superior, Universidad de Zaragoza, Ctra. Cuarte s/n, 22071 Huesca, Spain; 5 Cooperativa de Productores de Semillas de Arroz, Avenida del Mar 1, 46410 Sueca, Spain; 6 Centre Nacional d’Anàlisi Genòmica - Centre for Genomic Regulation (CNAG-CRG), Barcelona Institute of Science and Technology (BIST), Baldiri Reixac, 4, 08028 Barcelona, Spain; 7 Universitat Pompeu Fabra (UPF), Barcelona, Spain

**Keywords:** SNPs, *Oryza sativa*, Infinium SNP genotyping array

## Abstract

**Background:**

After its domestication, rice cultivation expanded from tropical regions towards northern latitudes with temperate climate in a progressive process to overcome limiting photoperiod and temperature conditions. This process has originated a wide range of diversity that can be regarded as a valuable resource for crop improvement. In general, current rice breeding programs have to deal with a lack of both germplasm accessions specifically adapted to local agro-environmental conditions and adapted donors carrying desired agronomical traits. Comprehensive maps of genome variability and population structure would facilitate genome-wide association studies of complex traits, functional gene investigations and the selection of appropriate donors for breeding purposes.

**Results:**

A collection of 217 rice varieties mainly cultivated in temperate regions was generated. The collection encompasses modern elite and old cultivars, as well as traditional landraces covering a wide genetic diversity available for rice breeders. Whole Genome Sequencing was performed on 14 cultivars representative of the collection and the genomic profiles of all cultivars were constructed using a panel of 2697 SNPs with wide coverage throughout the rice genome, obtained from the sequencing data. The population structure and genetic relationship analyses showed a strong substructure in the temperate rice population, predominantly based on grain type and the origin of the cultivars. Dendrogram also agrees population structure results.

**Conclusions:**

Based on SNP markers, we have elucidated the genetic relationship and the degree of genetic diversity among a collection of 217 temperate rice varieties possessing an enormous variety of agromorphological and physiological characters. Taken together, the data indicated the occurrence of relatively high gene flow and elevated rates of admixture between cultivars grown in remote regions, probably favoured by local breeding activities. The results of this study significantly expand the current genetic resources available for temperate varieties of rice, providing a valuable tool for future association mapping studies.

**Electronic supplementary material:**

The online version of this article (doi:10.1186/s12284-016-0130-5) contains supplementary material, which is available to authorized users.

## Background

Rice is a major crop with an enormous economic impact worldwide and it is widely cultivated throughout both tropical and temperate regions (Lu and Chang 1980). Modern rice (*Oryza sativa* L.) domestication occurred in southern China and, concomitant with human migrations, expanded to a wide range of geographical regions with diverse climates (Gross and Zhao 2014). As a consequence, it was generated an extensive and vast array of genetic diversity that in principle can be predominantly structured in two main subgroups (Childs 2004), including the *indica* and *japonica* varietal groups. These genetic groups are characterized by adaptations to specific climates, according to the agro-ecological conditions where they were cultivated. *Indica* genotypes are grown exclusively in tropical latitudes, whereas *japonica* genotypes can be found either in tropical or temperate climates (Mackill and Lei 1997).

Rice yield is highly influenced by cultivation practices in addition to climatic conditions. The adaptation process to new climates involved the selection of plants carrying genomic features that conferred advantages against adverse upcoming growth conditions that were transmitted through generations. During the northwards expansion of rice until the boundary limited by cold temperatures, crop adapted to new photoperiod conditions: the permissive summer temperatures with long days and short nights. While low temperature stress remains as the pivotal limitation in rice production in temperate regions (Andaya and Tai 2006), the acclimation to long day conditions of northern rice cultivars represents one of the main constrains during their expansion, and the most evident difference with the cultivars that remained in tropical latitudes (Izawa, 2007). Cultivation and breeding for centuries in diverse agro-ecological conditions gave rise to a myriad of different rice varieties that show the highest performance in the specific region where they were developed. The adaptations involved in this process are modifications in the regulation of metabolic and physiological processes that decrease plant yield and performance when grown out of their appropriated growth conditions. In this context, genes involved in flowering regulation should show allelic differentiation across environmental gradients, and their allele frequencies may reflect the mechanism of adaptation of plants to new conditions of day length through a geographic correlation pattern (Naranjo et al. 2014). In this regard, a significant intersubspecific variation concerning tolerance to cold stress could also be observed as the growing area approaches the northern limit (Baruah et al. 2009) and recently Ma et al. (2015) have described the COLD1 locus which is involved in the acclimation to cold of *japonica* rice. As a consequence of this intense and long-term breeding process, rice cultivars became adapted to specific regions around the world narrowing in this way its genetic pool, since many traits were forsaken due to the lack of interest in a certain moment. Nowadays it is difficult to reincorporate these characters because of the genetic distance raised between cultivars from tropical regions and those cultivated in temperate regions. The use of non-adapted varieties in breeding programs is challenging, as the incorporation of a new interesting trait is generally accompanied by many undesirable characters that do not meet climate adaptation requirements and consumers’ preferences.

In contrast with this generalized narrow genetic pool, the region where temperate *japonica* varieties are cultivated is wide enough to hold relevant natural diversity uncovering a wide spectrum of morphological and physiological variations. The characterization of this diversity, especially that concerning agronomic traits, constitutes the basis for genetic association analyses. Identifying loci that underlie this phenotypic variation is crucial for breeders, since it will offer opportunities to incorporate new traits of interest into local cultivars while conserving in unison those characters responsible for photoperiod adaptation.

The characterization of genome diversity can be efficiently performed by using next-generation sequencing (NGS) technologies that enable the massive identification of single nucleotide polymorphism (SNP) markers. These markers have been successfully applied for this purpose previously in many major crops (e.g. Myles et al. 2010) including rice (e.g. Huang et al. 2012; Xu et al. 2011). Databases of SNPs have been developed from the sequencing of numerous accessions of cultivated rice and wild rice (Duitama et al. 2015; McCouch et al.; 2016 Xu et al. 2011). The large-scale SNP database available at IRIC portal (http://oryzasnp.org/iric-portal/), for instance, has been generated as a result of re-sequencing 3000 rice genomes (3K RPG 2014). This huge amount of information constitutes an invaluable tool for breeders. However, breeding activities concern local varieties adapted to specific agro-climatic conditions, and therefore they should be complemented by studying the genetic variability within these local varieties to identify specific alleles that may introduced improvements by combination. In this study, we aimed the identification and characterization of high density SNP markers using NGS techniques in a collection of 217 rice varieties encompassing modern elite and old cultivars, and traditional landraces mainly cultivated under long-day photoperiod conditions. This collection attempts to represent the genetic diversity available for rice breeders, including traits that have been abandoned because of the continuous selection pressure and adaptation to changing agronomic environments and consumer demand conditions.

## Results

### Selection of 14 Cultivars Representative of the Genotypic Diversity of the Collection

**Fig. 1:**
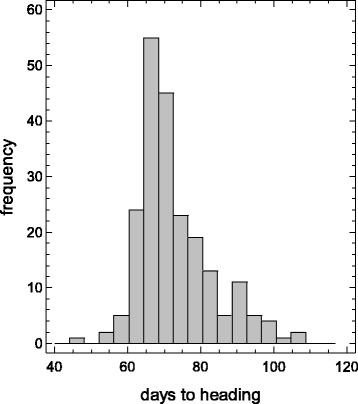
Distribution of the mean flowering time in the *japonica* rice core collection grown under natural long day conditions

A collection of 217 selected varieties was generated in order to analyse the population structure of rice grown under long photoperiod conditions. The collection was composed of modern and old cultivars as well as some landraces to cover a wider genetic diversity (Additional file 1: Table S1, Additional file 2: Figure S1). In this compilation (26 countries, 52.5 % arising from Europe; Additional file 1: Table S1), the core collection was composed of cultivars considered as *japonica* type from different geographical origins and cultivated in temperate climates in northern latitudes. A set of *indica* cultivars was also included in the collection as a reference of genetic divergence. The heading period of the varieties included in the collection ranged from 48 to 107 days (Fig. 1) and the longer periods corresponded to *indica* cultivars. Furthermore, one *indica* variety, Nona Bokra, failed at flowering in our growing conditions.

From this collection, 14 cultivars which covered the neutral genotypic diversity useful for breeding were selected (Table 1). Cultivars were selected according to their known genealogical data, temperature and photoperiod requirements and grain type.

**Table 1:** Country of origin, main traits interesting for breeders and sequencing statistics of 14 rice cultivars selected for genome sequencing in this study. Reads were mapped on the sequences of Nipponbare Os-Nipponbare-Reference-IRGSP-1.0. Number of reads, % of unique and duplicated reads and resulted mean coverages are reported. Number of total SNPs and number of total SNPs after filtering according to prediction significance, type and number of alleles and absence of repetitive sequences

Accession	Origin	Characteristic	Number of reads (× 10^3^)	% unique reads	% duplicated reads	Mean coverage	SNPs vcf	SNPs filtered
Arroz da Terra	Portugal	Cold tolerance, earliness	80,492	78.9	0.14	39.9	465,830	117,170
Bahia	Spain	Parental, grain quality	78,446	78.2	0.20	38.3	341,451	84,321
Bomba	Spain	Landrace, grain quality	80,038	77.0	0.12	38.7	771,460	200,619
Gigante Vercelli	Italy	Blast resistance	69,031	78.1	0.09	33.8	592,763	111,351
Gleva	Spain	Grain type	78,505	78.6	0.12	38.7	564,829	141,363
Italica Livorno	Italy	Earliness, cold tolerance	72,919	79.7	0.20	36.3	404,125	91,806
Kalao	France	Blast resistance	75,052	76.9	0.13	36.4	1,025,081	253,553
L202	USA	Parental, grain type	78,854	77.1	0.14	38.3	989,104	269,476
Loto	Italy	earliness	71,612	78.2	0.19	35.1	527,474	106,014
LTH	China	Cold tolerance	77,667	77.7	0.10	37.8	573,944	149,950
M202	USA	Parental	69,364	79.1	0.14	34.5	669,310	124,931
Pavlovski	Russia	Earliness, grain type	66,962	80.1	0.16	33.6	396,196	69,894
Puntal	Spain	Grain quality	69,581	77.6	0.17	34.0	928,794	182,272
Senia	Spain	Grain type	81,376	79.7	0.16	40.6	358,900	100,783

### Genome Sequencing and Identification of Polymorphisms

#### SNP panel for diversity of rice cultivated in temperate regions

Genome sequencing of the subset of 14 varieties generated a mean of 75 × 10^6^ short reads per cultivar that were mapped onto the Nipponbare reference genome (IRGSP-1.0). Approximately 78 % of them corresponded to unique reads (Table 1) while the mean coverage of these was x36. Comparison of the sequences with the reference genome provided a relatively high number of polymorphisms between the genomes analysed. Data were filtered according to different criteria as prediction significance, type and number of alleles, and absence of repetitive sequences. An average of 143,107 SNPs per genome were identified in the 14 genomes (Table 1), evenly distributed across the 12 chromosomes (Additional file 3: Table S2), and the number of non-redundant SNPS was 763,021.

#### SNP Panel for Analysing Genetic Diversity of *Japonica* Rice Cultivated in Temperate Regions

A custom Infinium SNP genotyping array (Illumina) compiling 2697 SNPs selected out of the polymorphisms identified in the 14 rice cultivars was designed. The SNP panel was generated by selecting bi-allelic polymorphisms with a uniform distribution along the 12 chromosomes, but avoiding centromere regions where gene occurrence is scarce. SNPs that were detected in more than one cultivar were prioritized. These selected SNPs were manually curated using Integrative Genomics Viewer (IGV, Broad Institute) software. The mean interval distance between adjacent SNPs was 137,525 pb. The number of SNPs per chromosome ranged from 163 in chromosome 9 (the shortest one, 23.0 Mb) to 321 SNPs in chromosome 1 (the largest, 43.2 Mb). The distribution of these SNPs reflected their non-redundancy. The number of selected SNPs varied among cultivars, ranging from 761 SNPs in L-202 to 502 in Bahia, with an average of 607 SNPs per genome (Table 2).

**Table 2:** Number of SNPs per chromosome selected in each rice cultivar and number of non-redundant SNPs. The Nipponbare genome was used as the reference genome

	Arroz da Terra	Bahia	Bomba	Gigante Vercelli	Gleva	Italica Livorno	Kalao	L-202	Loto	LTH	M-202	Pavlovski	Puntal	Senia	Total non-redundant SNPs
chr01	81	96	61	87	76	89	101	105	65	56	95	58	108	77	321
chr02	71	54	36	61	53	62	57	55	42	45	50	60	61	55	259
chr03	60	49	56	56	34	54	74	74	63	50	38	44	65	48	281
chr04	53	34	63	56	35	42	65	71	56	64	44	45	56	41	248
chr05	42	45	53	49	54	50	71	74	63	53	60	50	66	59	218
chr06	52	37	52	52	52	69	52	55	57	55	37	48	62	53	225
chr07	61	27	54	39	25	48	64	57	59	53	50	46	34	23	199
chr08	54	28	60	53	59	49	63	69	45	46	61	28	62	30	211
chr09	39	16	41	39	19	38	49	52	17	37	19	27	44	16	163
chr10	34	46	58	42	43	31	41	41	48	36	45	35	52	44	179
chr11	35	32	44	40	27	31	45	47	27	39	39	38	45	24	200
chr12	38	38	52	59	55	49	59	61	34	45	50	24	64	41	194
Total	620	502	630	633	532	612	741	761	576	579	588	503	719	511	2.698

**Fig. 2:**
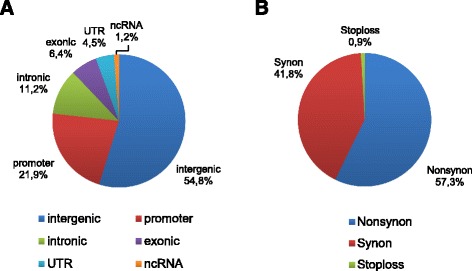
**a** Distribution of SNPs according to the TIGR gene annotation. Genic SNPs were classified as exonic, intronic and UTR SNPs. SNPs distributed within 2.0 Kb upstream of the coding region were identified as promoter SNPs. Nuclear RNAs (ncRNA) are also represented. **b** Distribution of exonic SNPs causing synonymous (synon) or nonsynonymous (nonsynon) substitutions or producing changes in the stop codon (lossstop)

The proportion of SNPs located within intergenic regions (54.8 %) was higher than those within genic regions (22.1 %) (Fig. 2a). Genic SNPs were classified as exonic (6.4 %), intronic (11.2 %) or UTR (4.5 %), while the proportion of SNPs distributed within promoter regions was similar to that of genic SNPs (21.9 %). Among coding SNPs, nonsynonymous substitutions (57.3 %) were higher than synonymous substitutions (41.8 %) (Fig. 2b). Meanwhile, SNPs causing large effect thus affecting the integrity of encoded proteins were not frequent, and only 0.9 % of SNPs causing disruption of the stop signal were detected.

**Fig. 3:**
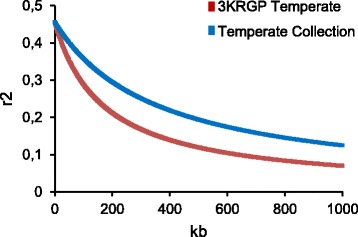
Estimated LD decay from the *japonica* subpopulation in the collection and the 3KRPG *japonica* subpopulation, expressed as decay of r^2^

Then, 217 rice accessions were genotyped with this array of 2697 SNP and a genetic profile was obtained for each cultivar. Comparison between the genetic profiles allowed the identification of low frequency alleles and those with a minimum allele frequency (MAF) below 5 % were removed, then originating a panel of 1713 SNPs evenly distributed along the genome at a mean distance of 215,223 pb. Using this panel we estimated the extent of linkage disequilibrium (LD) in the *japonica* subpopulation in the collection, and compared it with that estimated for the temperate *japonica* subpopulation in the 3KRPG (http://oryzasnp.org/iric-portal/). The LD decay rate was calculated as the chromosomal distance where the correlation coefficient (*r* ^*2*^) between SNP pairs dropped to half its maximum estimated value. LD estimation reflects the strong population structure of the *japonica* subpopulation in our collection, since *r* ^*2*^ drops to 0.23 when chromosomal distance extends to approximately 368 kb while in the 3KRPG subpopulation we estimated a more rapid decay that extends to 174 kb for the same value of correlation coefficient (Fig. 3).

**Fig. 4:**
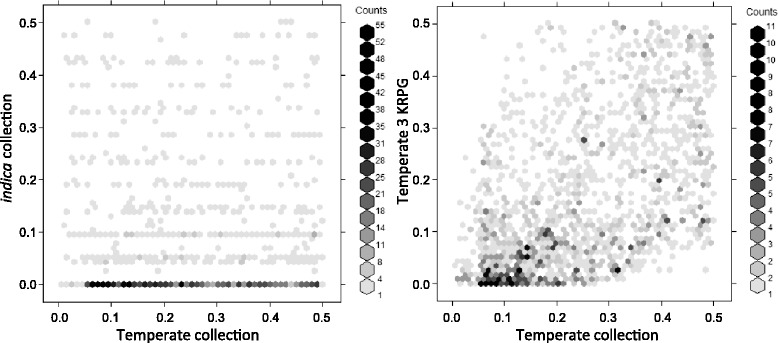
Allele frequencies in the different subpopulation. Two-dimensional site frequency spectra for (left panel) both temperate *japonica* subpopulations in the collection vs 3 K RPG and (right panel) temperate *japonica* vs *indica* subpopulations in the collection

To further explore the adequacy of the SNPs array for the analysis of the population structure, we examined 2D site frequency spectra between *japonica* and *indica* varietal groups in our collection and in the temperate *japonica* 3KRPG subpopulation (Alexandrov et al. 2015). We found that the majority of SNPs that are at high frequency in *japonica* cultivars in the collection are found at markedly lower frequencies in *indica* cultivars (Fig. 4) meanwhile the majority of SNPs that are at low frequency in *japonica* cultivars in our collection are also found at low frequency in *japonica* 3 K RPG subpopulation (Fig. 4).

### Genetic Structure of the Collection

**Fig. 5:**
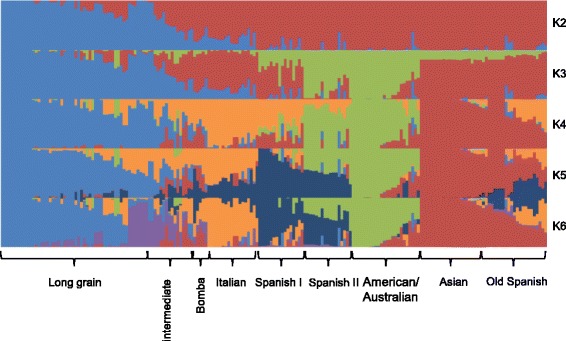
Population structure of the collection of *japonica* rice cultivars. The figure shows the estimated membership probability of assigning cultivars of the collection to either 6 (**a**) or 9 (**b**) subgroups. Bar length represents the probability of each variety to belong to different subgroups. The cultivars were sorted according to their membership probability in (**a**)

To determine the population structure of the collection, a 948 SNP panel based on the above LD decay results and with a mean interval distance of 390,228 pb was designed. The most probable number of subpopulations, and cultivars included in each, was estimated using STRUCTURE software. According to theses analyses, ∆*K* showed maximum values for K = 4 (∆*K* = 101.2) indicating that the optimum number of subpopulations was 4 (Fig. 5, Additional file 1: Table S1). Long grain type accessions conformed subpopulation 2 and displayed different geographical origins from Europe, America and Australia. Medium grain type cultivars were distributed in different subpopulations in accordance with their geographical origin: cultivars in group 4 were mainly from Italy, while cultivars in group 3 originated in America, Spain and Australia. Most of the Spanish cultivars included in group 3 were of recent release, while some old accessions from Spain and Italy were included in group 1, in which Asian *japonica* cultivars were clustered, thus revealing the putative Asian ancestry of these European old varieties. Differentiation of these four subpopulations was corroborated by the high Fst values obtained for each group although the fourth genetic group, composed mainly by Italian accessions, showed the lowest value of genetic differentiation (Additional file 4: Table S3, Additional file 5: Table S4).

A further maximum of ∆K was found for K = 5 (∆*K* = 1.8), which separated a new subgroup that included Spanish cultivars. Accessions showing higher than 80 % of membership to this cluster were released in the mid-twentieth century. A total of 12 accessions were classified as admixed and showed approximately 50 % of membership to the Spanish and American clusters. This admixed group includes accessions originated in Spain that were released approximately at the same time, during the second half of the twentieth century. The proportion of the genetic content belonging to these two clusters clearly reflects their breeding history, since these cultivars were obtained when American germplasm was introduced into the Spanish breeding programs in the early 20th century.

A subsequent division of long grain cluster was observed at K = 6 (∆K = 3.5), which separated in a new subgroup a set of long grain varieties, as Moroberekan, Agami and Honduras, cultivated in tropical regions.

**Fig. 6:**
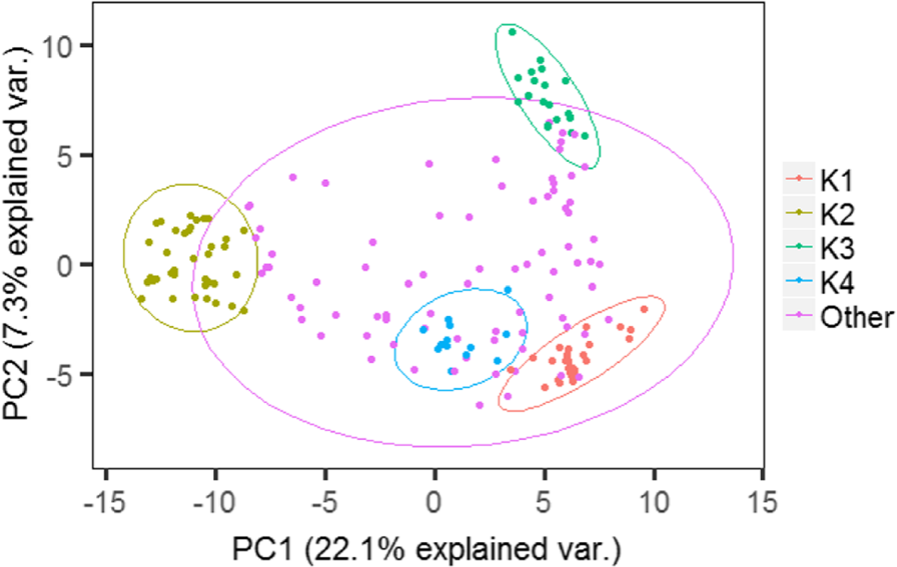
Principal components analysis (PCA) plots from 1713 SNP panel of the collection of *japonica* rice cultivars. First and second principal components are shown and the proportion of the variance explained by each principal component is indicated in parenthesis. Colors refer to *k* = 4 genetic groups with a membership above 80 % as showed in Fig. 5 (see Bayesian structuring results)

To deepen in the patterns of population structure we performed principal components analysis (PCA) with the set of 1713 SNPs selected from the Infinium analysis. The first PC, which accounted for 22.1 % of the variance, separated cultivars according to the grain size, which agrees with the four most probable groups described in the population structure analysis, since long-grain type cultivars included in cluster 2 are separated from the other groups (Fig. 6).

### Genetic Relationships Among the Temperate Rice Collection

**Fig. 7:**
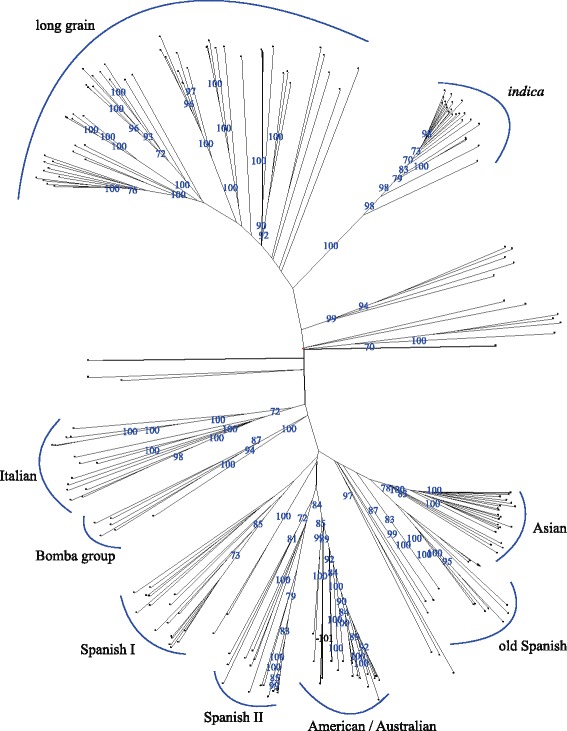
Neighbour-Joining tree of 217 rice accessions based in Sokal & Michener distance as implemented in Darwin 6.0. Values in branches indicate the bootstrap (10,000 replicates) support values

The 1713 SNPs panel was used to determine the relationship of cultivars in our collection and to estimate genetic distances among them. Basically, the 217 cultivars were grouped into a dendrogram with clusters arranged similarly to the distribution obtained in the STRUCTURE analysis (Fig. 7, Additional file 6: Figure S2). The distribution of cultivars obtained was roughly in accordance with their origin or grain type. On one side of the tree, *indica* varieties appear in a highly supported cluster. Closely related to *indica* cluster, long grain type varieties diverge into a wider cluster that includes most of the varieties grouped in cluster 2 in the population structure analysis, such as *japonica* accessions from tropical regions (Azucena or Moroberekan) and temperate regions (Cormoran or Apolo). Aromatic rice cultivars, for instance Fragance or Giglio, can also be distinguished among them.

All the remaining varieties, mostly medium-grain type, are genetically distant from the long-grain and *indica* clusters. The medium-grain varieties were grouped in 7 different clusters with a remarkable influence of the geographic origin (Fig. 7). Several European landraces were clustered together within a wider cluster that includes Asian representatives. The use of a higher number of SNPs to construct the dendrogram allowed the fragmentation of European cultivars into several small subgroups, with a distribution that clearly matches its origin and, even from some occasions, the release date of the cultivars. Asian cultivars are clearly related to a cluster formed by ancient Spanish varieties, revealing the influence of Asian germplasm in the early reported history of cultivars grown in Spain. Another two clusters are constituted by Spanish varieties, which are closely related to the American/Australian group, indicating the relevance of the introduction of American germplasm in the Spanish breeding programs. This genetic relationship was also observed in the population genetic analysis. Finally, a fourth cluster of Spanish landraces grouped together the five accessions with Bomba type grain, which possesses specific culinary qualities, emerge as an independent cluster.

## Discussion

The expansion of rice to a wide geographical area after its domestication generated enormous diversification due to the progressive breeding activity through the selection of plants that showed superior performance under the different local climate conditions leading to a broad range of novel phenotypes. Genetic diversity is a natural resource for rice breeding to meet current food demands. Understanding population structure and genome variations are crucial achievements to facilitate genome-wide association studies of complex traits and functional gene investigations. In this regard, our study provides a useful pool of SNP markers with deep coverage throughout the rice genome. We have characterized 1713 SNPs that were genotyped in 217 rice varieties. Cultivars were representative of all genetic groups found across geographical regions in temperate climate as revealed by our population structure analysis and in accordance with previous studies (Courtois et al. 2012). Our genomic diversity and structure analyses have included old and modern *japonica* cultivars attempting to uncover a maximum spectrum of variability. This allowed us to reconstruct the genetic relationships and genetic diversity among several temperate rice varieties from distant geographic origins (Additional file 1: Table S1) which possess an enormous variability in agro-morphological and physiological traits.

The use of a high number of SNP markers, provided by deep sequencing of a selected group of cultivars, enabled us to analyse in detail the temperate *japonica* group, and in particular, the genetic relationship of several Spanish cultivars with other groups from different countries. This allowed us to revisit the history of rice breeding in Spain. As previous genetic studies in rice (Huang et al. 2010; Xu et al. 2011; Zhao et al. 2010), our analysis showed strong genetic substructuring within *japonica* accessions. This is in accordance with the enlarged LD decay observed in the collection compared to other reported small subpopulations (Xu et al. 2011), that can be an effect of the sampling composition and size. One of the relevant observations in our analysis is the influence of the grain type to the genetic classification of cultivars, suggesting that this trait is a pivotal factor in the morphological structure of current and old rice varieties. Accordingly, the dendrogram based on genetic distances establishes clear-cut differences among long and medium type grain cultivars with independence of their origin (Fig. 7). This pattern was also observed in the PCA analyses, which separated both types of cultivars in the first axis (Fig. 6). The origin of long grain varieties does not appear to be a discriminating factor among this group that certainly contains many varieties from several continents. Thus, this cluster includes cultivars grown in tropical regions (e.g. Philippines or Honduras), as well as in countries with temperate climate, as European countries. Furthermore, cultivars in the collection considered as tropical *japonica*, such as Azucena, Honduras, Katy and Lemont (Zhao et al. 2010), are located within the long grain cultivars.

On the other hand, several subpopulations of *japonica* varieties with medium grain size can be distinguished in different secondary branches. In this case, accessions from nearby locations were more closely related than samples from distant locations, showing geographic patterns of genetic variation probably produced by breeding and selection associated with local food preferences.

Spanish varieties, the most numerous group in our population, are grouped into four clusters that are predominantly related to the time they were cultivated and that clearly reflect the history of rice breeding in Spain. The Bomba family, consisting of the most ancient Spanish landraces and whose origins precede the available records, appeared in a distinct and isolated cluster in the dendrogram. Some of these cultivars, well appreciated by consumers, are still cultivated because of the peculiar characteristics of their grains and because of their agronomic characteristics that are suitable for organic farming. A second group of Spanish cultivars derived from the earliest breeding activities in Spain, remains in a distinct group close to the Asian cluster, indicating the origin for the first cultivars used as donors in initial breeding programs. In a third separate cluster we found varieties that were grown in Spain during the first half of the 20th century. This cluster appears very close to the node that separates the American-Asian clusters. Modern Spanish cultivars are located in a secondary cluster nested to the America-Australian variety group. These results, together with the genetic admixture suggested by the Bayesian structuring analyses with a similar membership probability to the American and Spanish subgroups when *K* = 5, suggest the introduction of American germplasm varieties in the Spanish breeding program.

Previous genetic studies pointed to a narrower genetic diversity in *japonica* than in *indica* rice, probably due to a more severe domestication bottleneck (Garris et al. 2005). Additionally, breeding activities during the last century also constricted the genetic pool of cultivars selected for cultivation in different agro-ecological environments. In that sense, searching for new potential donors for breeding activities has become a difficult task since the use of non-adapted donors from other latitudes to introduce particular traits entails accompaniment of characters that may not be agronomically appropriate in the region. This is the case of the hybridization between temperate and tropical *japonica* cultivars. In addition, high sterility has been observed in *indica*-*japonica* hybrids due to strong reproductive barriers which hampered gene flow between them (Jeung et al. 2005). On the contrary, a gene flow occurred between cultivars grown in temperate regions as revealed in the Spanish breeding history deduced from our genetic analysis, showing the influence of various genetic groups in the Spanish cultivars. During the beginning of breeding activities, genetic influence from Asia was high in medium- grain rice and some links with Italian cultivars were evident. This influence diminished as new improved varieties were generated, showing increasing membership to clusters composed by American cultivars, in detriment of Italian accessions.

Natural diversity distributed across different geographical regions during the expansion of rice constitutes a source of genetic variability highly valuable for the breeding programs to generate new varieties that are adapted to local climate conditions. Breeders need the natural genetic resources to recover agronomic traits of interest that have been left behind during the selection process, but knowledge of the chromosomal regions responsible for many phenotypic variations remains undiscovered. In this context, association mapping and genomic selection are recently developed methodologies that have been proved to be effective approaches to connect structural genomics and phenotype and, thereby, to mine elite genes in germplasm resources (e.g. Zhang et al. 2014; Begum et al. 2015). Our highly variable SNP set among and between several temperate rice varieties constitutes a short-term tool for exploring candidate genes involved in several traits related to the adaptation to temperate climate as heading time or cold tolerance. Rice cultivation in temperate regions deals with low temperature stress as a main yield limitation (Andaya and Tai 2006) since rice varieties grown under temperatures below 15 °C usually suffer from low germination rates, yellowing or withering, reduced tillering, delayed heading or sterility (Kaneda and Beachell 1974; Mackill and Lei 1997; Yoshida et al. 1996). Some *japonica* accessions, for instance, have been characterized and selected for cultivation in temperate regions because of their high tolerance to low temperature while producing good grain quality and high yield (Fujino et al. 2004; Jiang et al. 2008). Therefore, identifying cultivars closely related to cold tolerant ones constitutes the basis to carry out breeding programs based on recombinant inbred activities.

## Conclusions

The analysis of the genomic profile of 217 rice varieties revealed the genetic distances and diversity among rice cultivars grown in temperate climate regions. Our observations indicated a contribution of the grain type to the population genetic structure stronger than that of the geographic origin of temperate *japonica* cultivars. The use of a high number of SNP markers in this study also revealed gene flow and higher rates of admixture between cultivars grown in remote regions, probably as a consequence of local breeding activities. We could also observe the influence of Asian and American cultivars at the early and the most recent steps respectively, of rice breeding in Spain.

## Methods

### Plant Material and Growing Conditions

The rice core pool was obtained from different germplasm collections: the International Rice Research Institute (IRRI, Philippines), U.S. National Plant Germplasm System (NPGS, USA), Rice Genome Resource Center (RGRC, Japan) and Instituto Valenciano de Investigaciones Agrarias (IVIA, Spain). Seeds of different cultivars were germinated and grown in pots in greenhouses (39° 28’ N) under controlled temperature (25 °C) and relative humidity (50 % RH), and in natural daylight conditions during summer in 2013 and 2014. Plantlets were manually transplanted in rows of 20 plants in May and harvested in September. Fields were irrigated by flooding. Heading time was measured as 50 % of panicle emergence in each row.

### Whole Genome Sequencing

Fourteen varieties representative of *japonica* cultivars were previously selected to carry out the SNPs characterization (Table 1). Seven days-old seedlings were grown in the dark for 2 days and then nuclear genomic DNA was extracted from leaves by using a modified CTAB protocol (Schneeberger et al. 2009). Genome sequencing was performed at the Centro Nacional de Análisis Genómico (Barcelona, Spain) as follows: the short-insert paired-end libraries were prepared with NO-PCR protocol. TruSeq™DNA Sample Preparation Kit v2 (Illumina Inc.) and the KAPA Library Preparation kit (Kapa Biosystems) were used. In short, 2.0 micrograms of sheared genomic DNA was end-repaired, adenylated and ligated to Illumina specific indexed paired-end adaptors. The DNA was size selected with AMPure XP beads (Agencourt, Beckman Coulter) in order to reach the fragment size of 220-550 bp. The final libraries were quantified by Library Quantification Kit (Kapa Biosystems).

The libraries were sequenced using TruSeq SBS Kit v3-HS (Illumina Inc.), in paired end mode, 2x101bp, in ½ of a sequencing lane of HiSeq2000 flowcell v3 (Illumina Inc.) according to standard Illumina operation procedures with the yield of >14Gb and median coverage of 33-41x. Primary data analysis, the image analysis, base calling and quality scoring of the run, was processed using the manufacturer’s software Real Time Analysis (RTA 1.13.48) and followed by generation of FASTQ sequence files by CASAVA. Data have been deposited at the European Nucleotide Archive (ENA) in the European Bioinformatics Institute (EBI) with the accession number PRJEB13328 (http://www.ebi.ac.uk/ena/data/view/PRJEB13328).

### Sequencing Data Analysis

Reads were hard trimmed from the end up to the first base with a Phred quality of at least 10. Reads with a length of at least 40 nt were mapped to the Nipponbare rice variety reference genome (the unified-build release Os-Nipponbare-Reference-IRGSP-1.0 (IRGSP-1.0)) using the GEM toolkit (version 2) (Marco-Sola et al., 2012) allowing up to eight mismatches per read. Only uniquely mapping non-duplicate read pairs were used for subsequent analyses. The SAMtools suite (version 0.1.18) (Li et al. 2009) with default settings was used to call SNVs and short INDELS per variety. Genome annotations from http://rapdb.dna.affrc.go.jp/download/irgsp1.html such as genes and CDS were added to the resulting VCF using vcftools (Danecek et al. 2011). Variants identified in regions with low mappability, with a pronounced strand bias with a *p*-value of <0.001 or a pronounced tail distance bias with a *p*-value of < 0.05 were filtered out.

### SNP Panel and Genotyping

A panel of 2697 SNPs representative of *japonica* rice cultivated under long day conditions was generated by selecting polymorphisms identified in the 14 cultivars. Bi-allelic polymorphisms with a uniform distribution along the 12 chromosomes SNPs were selected, avoiding centromeric and telomeric regions and maintaining an average distance of 137.6 kb among them. SNP polymorphisms present in more than one cultivar were prioritized. SNPs were visually inspected using Integrative Genomics Viewer software (IGV, Broad Institute). The adaptability to the Illumina iSelect detection system of the SNPs and their neighbouring sequences was scored and SNPs with a score higher than 0.4 were selected for the multiplexing Infinium Assay (Illumina Inc.).

The 217 accessions were genotyped with the 2697 SNP array. Comparison of the called genotypes was performed using GenomeStudio software (Illumina). Low frequency alleles, considered when they were present in less than 5 % of the cultivars, were removed. Genotypes were missing data if they failed in more than 50 % of the cultivars.

Genomic variation analysis and classification of SNP according to the TIGR rice gene models was implemented in platform CARMO (Comprehensive Annotation of Rice Multi-Omics data) (Wang et al. 2015, http://bioinfo.sibs.ac.cn/carmo/).

### Linkage Disequilibrium (LD) Estimation

Raw data was filtered to select 2066 markers with a minimum of genotype call of 75 % individuals and to eliminate monomorphic markers showing a MAF lower than 5 %. Linkage disequilibrium was calculated using Plink 1.07 (Purcell et al. 2007) according to Zhao et al. (2011). After filtering, the 1713 remaining SNPs were used to calculate LD using “--r2 --ld-window 99999 --ld-window-r2 0” commands. The decay of linkage disequilibrium by distance was fitted using Hill and Weir (Hill and Weir 1988) expectation of *r* ^*2*^ between adjacent sites. Calculations were performed according to the equation by Remington et al. (2001). Nonlinear least squares, implemented in the ‘nls’ R package, were used to fit equations to our data as suggested by Marroni et al. (2011).

### Site Frequency Spectra

Minimum allele frequency (MAF) for the 1713 SNPs panel in *japonica* and *indica* groups of our collection and for those from 3KRGP were calculated using Tassel (Tassel 5.2.26, Bradbury et al. 2007). In case of 3KRGP varieties, MAF was set after removing third state of SNPs and imputing by fillin. MAF bins values for each group of varieties were calculated using hexbin R package. The number of bins was set to 50. Since we used MAF, the values of allele frequency did not exceed 0.5.

### Principal Component Analysis

A Principal Component Analysis (PCA) was performed using the *prcomp* command in R version 3.2.3. We called genotypes for 1713 SNPs from the panel used in the LD analysis. Data were analyzed using *ggbiplot* (version 0.55).

### Estimation of Genetic Structure

Population genetic structure of the cultivars was estimated using the Bayesian clustering method implemented in STRUCTURE 2.3.4 (Pritchard et al. 2000). This approach estimated the optimal number of genetic clusters (*K*) and calculated the membership proportion of cultivars to them. Analyses were based on the admixture ancestral model for a range of *K* values from 1 to 15. We performed 20 runs for each *K*, and removed those with extreme values of L(*K*) that were tagged as outliners according to Evanno et al. (2005). Each run was implemented with a burn-in period of 100,000 steps followed by 1,000,000 Monte Carlo Markov Chain iterations. The optimal number of *K* clusters was estimated with the ad hoc parameter (Δ*K*) of Evanno et al. (2005) in Structure Harvester (Earl and vonHoldt 2012). We estimated the optimal alignment for the 20 replicates in CLUMPP (Jakobsson and Rosenberg 2007), employing greedy algorithm with 10,000 permutations. Cultivars were subdivided into different subgroups according to their maximum membership probability among the subgroups and the membership probabilities threshold of 0.80.

Pairwise genetic distances between rice cultivars were calculated as implemented in Darwin 6.0 (Perrier et al. 2003), by using the Sokal & Michener index and 10,000 bootstrap replicates. Then we obtained a neighbour-joining (NJ) tree, which robustness was assessed again by 10,000 replicate bootstrap analyses.

We also used the software STRUCTURE to estimate Fst values for the genetic clusters established for each group, the mean value of 20 runs was calculated. Finally, Fst pairwise values between the subpopulations defined by STRUCTURE analysis were calculated in Arlequin (Excoffier and Lischer 2010) with 10,000 permutations and including only those varieties with membership values > 80 %.

## Additional files

Additional file 1: Table S1. List of cultivars included in the analysis with their country of origin, days to heading and release date (a, before 1900; b, 1900–1965; c, 1966–2000; d, after 2000). Membership (>80 %) to K = 4, K = 5 and K = 6 groups defined by STRUCTURE is also indicated. Accessions displaying approximately 50 % of membership to two subpopulations are also indicated. (XLSX 21 kb)
Additional file 2: Figure S1. Geographic origins of cultivars used in the structure population analysis. (PDF 3794 kb)
Additional file 3: Table S2. Number of SNPs per chromosome present in the 14 selected cultivars referred to Nipponbare genome and percentage of SNP present in each chromosome in each cultivar. The number of non-redundant SNPs in each chromosome is indicated. (XLSX 15 kb)
Additional file 4: Table S3. Mean and standard deviation values of proportion of membership, Avdistance (expected heterozygosity) and Fst for the four genetic groups established by STRUCTURE. (DOCX 12 kb)
Additional file 5: Table S4. Arlequin software estimates of Population pairwise differences (Fst) among the four genetic groups established by STRUCTURE. (DOCX 11 kb)
Additional file 6: Figure S2. Neighbour joining tree of 217 rice accessions. Accessions names are shown. (PDF 1227 kb)
