# Supplementary figures and images for: Genetic Diversity and Population Structure of Rice Varieties Cultivated in Temperate Regions
Source: Rice (N Y). 2016 Oct 20;9:58. doi: 10.1186/s12284-016-0130-5 (PMC5073090; doi:10.1186/s12284-016-0130-5)

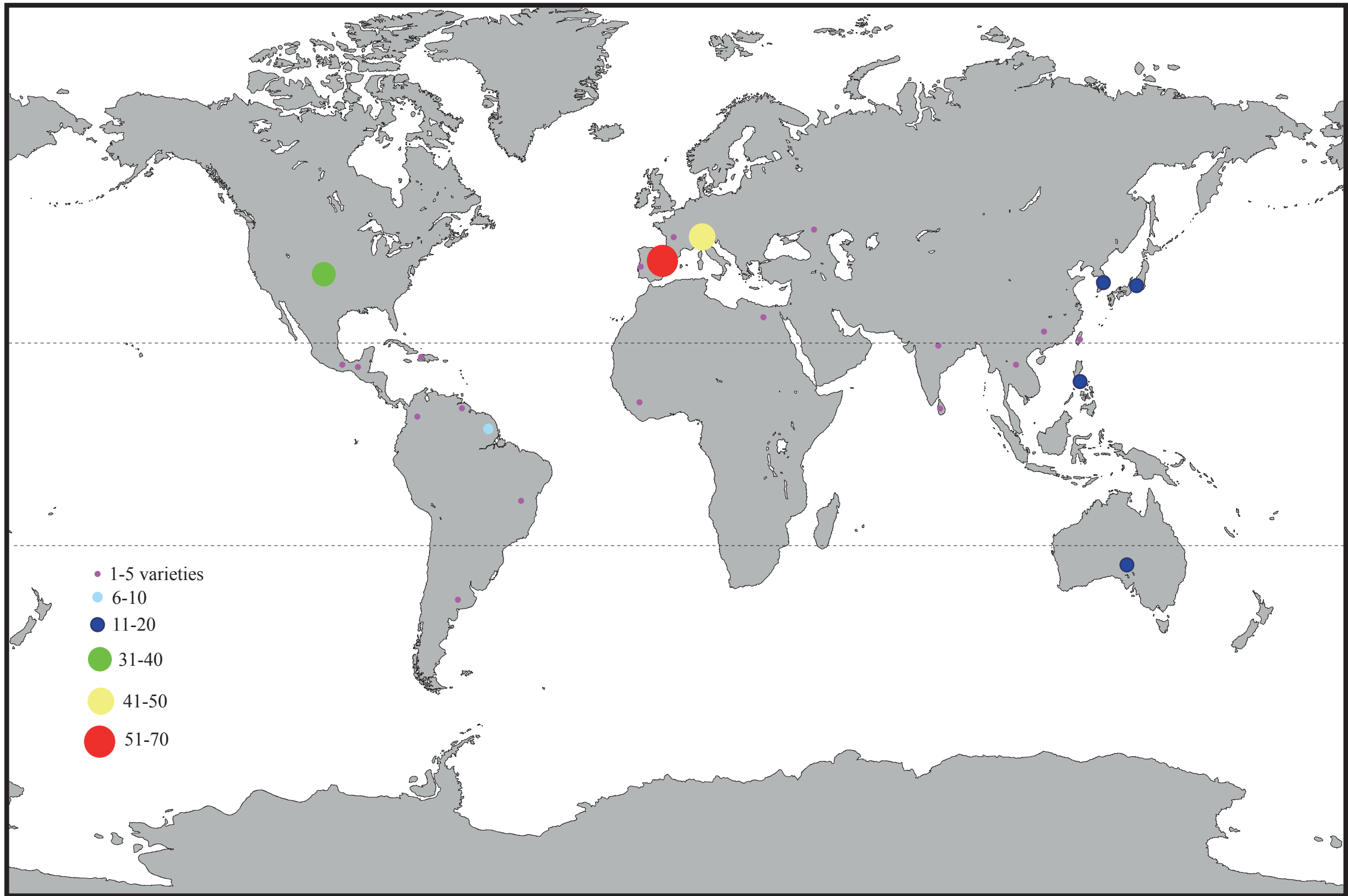

Supplement: Additional file 2: Figure S1. — Geographic origins of cultivars used in the structure population analysis. (PDF 3794 kb) [file 12284_2016_130_MOESM2_ESM.pdf]

long grain

aromatic

indica

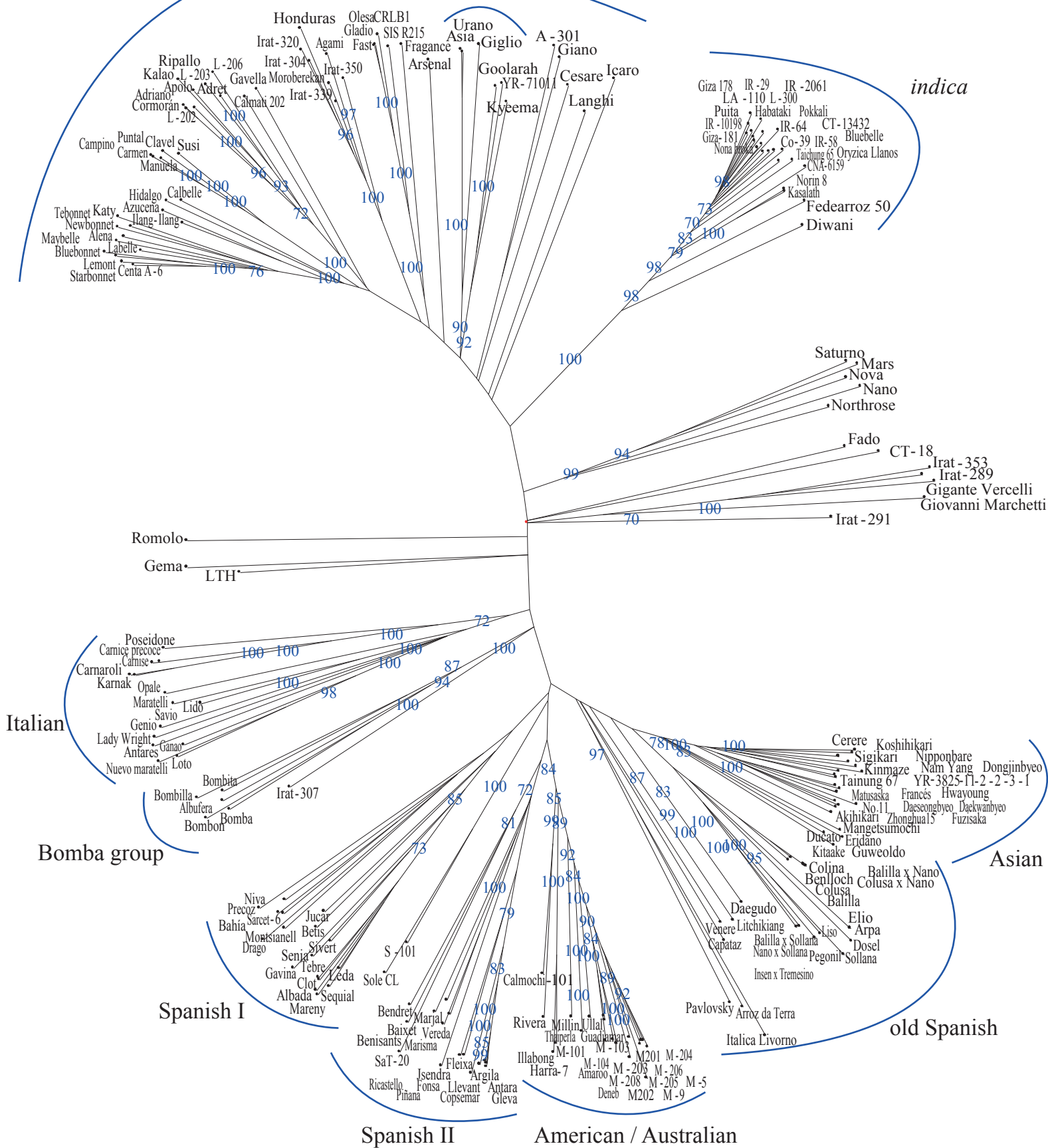

Supplement: Additional file 6: Figure S2. — Neighbour joining tree of 217 rice accessions. Accessions names are shown. (PDF 1227 kb) [file 12284_2016_130_MOESM6_ESM.pdf]
