# Supplementary material for: Genetic Diversity and Population Structure of Rice Varieties Cultivated in Temperate Regions
Source: Rice (N Y). 2016 Oct 20;9:58. doi: 10.1186/s12284-016-0130-5 (PMC5073090; doi:10.1186/s12284-016-0130-5)
Supplement: Additional file 4: Table S3. — Mean and standard deviation values of proportion of membership, Avdistance (expected heterozygosity) and Fst for the four genetic groups established by STRUCTURE. (DOCX 12 kb) [file 12284_2016_130_MOESM4_ESM.docx]

**Table** **S3**.- Mean and standard deviation values of proportion of membership, Avdistance (expected heterozygosity) and Fst for the four genetic groups established by STRUCTURE.

| Group | Mean proportion of membership | Mean Avdistance | Mean value of Fst |
| --- | --- | --- | --- |
| 1 | 0.264 ± 0.010 | 0.115 ± 0.001 | 0.728 ± 0.005 |
| 2 | 0.298 ± 0.007 | 0.172 ± 0.003 | 0.654 ± 0.009 |
| 3 | 0.195 ± 0.016 | 0.147 ± 0.023 | 0.683 ± 0.046 |
| 4 | 0.242 ± 0.020 | 0.259 ± 0.006 | 0.394 ± 0.084 |
