# Supplementary material for: Genetic Diversity and Population Structure of Rice Varieties Cultivated in Temperate Regions
Source: Rice (N Y). 2016 Oct 20;9:58. doi: 10.1186/s12284-016-0130-5 (PMC5073090; doi:10.1186/s12284-016-0130-5)
Supplement: Additional file 5: Table S4. — Arlequin software estimates of Population pairwise differences (Fst) among the four genetic groups established by STRUCTURE. (DOCX 11 kb) [file 12284_2016_130_MOESM5_ESM.docx]

**Table S4.-** Arlequin software estimates of Population pairwise differences (Fst) among the four genetic groups established by STRUCTURE

| group | 1 | 2 | 3 |
| --- | --- | --- | --- |
| 2 | 0.62476 |  |  |
| 3 | 0.49553 | 0.59288 |  |
| 4 | 0.45751 | 0.49343 | 0.45836 |
